# Age-related Eye Diseases: An Emerging Challenge for Public Health Professionals

**Published:** 2005-06-15

**Authors:** Christopher Maylahn, Dorothy M Gohdes, Appathurai Balamurugan, Barbara A Larsen

**Affiliations:** Division of Chronic Disease Prevention and Adult Health, New York State Department of Public Health; Association of State and Territorial Chronic Disease Program Directors, Albuquerque, NM; Diabetes Prevention and Control Program, Arkansas Department of Health, Little Rock, Ark; Association of State and Territorial Chronic Disease Program Directors, Santa Fe, NM

## Abstract

In April 2004, The Eye Disease Prevalence Research Group published a series of articles that included age-specific estimates for the prevalence of low vision and blindness in whites, African Americans, and Hispanics living in the United States. Also included were age-, sex-, and ethnic-specific incidences of the following age-related eye diseases: diabetic retinopathy, macular degeneration, cataracts, and glaucoma.

We reviewed the group's series of articles and highlighted key findings on the overall prevalence of and risk factors for age-related eye diseases, as well as opportunities to preserve and restore vision. We examined publications that show the public health impact of age-related eye diseases and the importance of projected increases in prevalence of low vision and blindness.

Approximately 1 in 28 Americans aged older than 40 years is affected by low vision or blindness. Among community-dwelling adults, the prevalence of low vision and blindness increases dramatically with age in all racial and ethnic groups. Whites have higher rates of macular degeneration than African Americans, but glaucoma is more common among older African Americans. Between 2000 and 2020, the prevalence of blindness is expected to double.

Age-related eye diseases are costly to treat, threaten the ability of older adults to live independently, and increase the risk for accidents and falls. To prevent vision loss and support rehabilitative services for people with low vision, it is imperative for the public health community to address the issue through surveillance, public education, and coordination of screening, examination, and treatment.

## Introduction

Age-related eye diseases (AREDs) that lead to low vision and blindness are a growing challenge for public health professionals (1). New estimates for the prevalence of AREDs and vision loss became available in 2004 from The Eye Disease Prevalence Research Group and showed that 1 in 28 Americans aged older than 40 years is blind or has low vision (2,3). Although treatments exist to prevent or delay vision loss for several of these conditions, the number of blind individuals is expected to double by 2020 as the U.S. population ages (2,3). In this review, we summarize the age, sex, and race prevalence rates for each of four AREDs — diabetic retinopathy, macular degeneration, cataracts, and glaucoma — and describe risk factors for the diseases. We also discuss the impact of AREDs and opportunities to prevent blindness, restore vision, and support people with visual impairments.

## Prevalence and Risk Factors for Age-related Eye Diseases

### Diabetic retinopathy

Of the AREDs, diabetic retinopathy is most familiar to public health professionals because of the efforts of state diabetes programs to increase eye examination rates among people with diabetes. Vision loss from diabetic retinopathy can result from several diabetes-related changes in the eye, but early recognition and treatment (including laser therapy) can prevent blindness (4,5). Treatment recommendations emphasize the need for aggressive blood glucose and blood pressure control to reduce the incidence, progression, and severity of diabetic retinopathy (6). Although a few studies have found a relationship between smoking and the risk of diabetic retinopathy progression, the conclusion of the most recent Surgeon General's report on the health consequences of smoking was that the evidence did not support a causal relationship between smoking and diabetic retinopathy (7).

In the United States, some form of diabetic retinopathy was detected in 40% of people with diabetes (8). Vision-threatening retinopathy was much less common, developing in 8% of people with diabetes. Because retinopathy depends on the duration of hyperglycemia rather than age, the actual prevalence of retinopathy among people with diabetes did not increase markedly when stratified by age (9). However, the prevalence of retinopathy in the general U.S. population older than 40 years was 3.4% and increased with age, reflecting the higher rates of diabetes in older persons (8).

### Age-related macular degeneration

Like diabetic retinopathy, age-related macular degeneration (AMD) has several forms (10,11). The form known as *wet AMD* is caused by abnormal vascularization under the retina and is an important cause of central vision loss. *Dry AMD,* the most common form, tends to progress more slowly than the wet form. The most advanced form, *geographic atrophy,* also causes central vision loss. Although the pathogenesis of AMD is not totally understood, 54% of the cases of blindness in white Americans have been attributed to the disease (2). AMD increases dramatically with age in men and women, but no significant sex difference in rates has been found (12). AMD is less common in African Americans than in whites. According to community eye studies, as many as 16% of white women and 12% of white men aged 80 years and older have advanced AMD.

Several pathophysiological processes associated with aging have been proposed as etiologies for AMD, and research on the topic continues (13). In one study, high-dose supplements of vitamins C and E, beta carotene, and zinc slowed the rate of progressive vision loss somewhat over 5 years, but only when the condition was not extremely advanced (14). The recognition of a link between high-dose supplements of beta carotene in smokers and increased cardiovascular risk may have discouraged eye-care professionals and patients from using the vitamin-supplement regimen found to be effective (15,16). However, it has been estimated that in 300,000 people aged 55 years and older, additional vision loss over the next 5 years can be prevented if the supplementation study results are translated into widespread clinical practice (17). Laser therapy and photodynamic therapy can be used successfully for some forms of wet AMD, although the disease is particularly complex and challenging to treat (10,11). New forms of therapy, such as injecting antivascular endothelial growth factor, effectively reduced the progression of vision loss in a randomized trial of people with advanced wet AMD (18,19). No surgical interventions for the dry form of the disease exist, although the efficacy of laser photocoagulation of the macula to prevent progression of early disease is being studied. Rehabilitative interventions for individuals who are in their 80s and 90s and have low vision because of AMD are challenging, but quality of life can be improved (20). Helping the growing number of older adults with low vision to function independently will tax the already strained vision rehabilitation resources in many communities.

### Cataracts

Cataracts are the most common ARED. In community studies, more than 17% of Americans aged 40 and older were estimated to have one or more cataracts, and 5% reported having had surgery to remove a cataract from one or both eyes (21). In 1991, 1.35 million surgical procedures to remove cataracts were performed among Medicare beneficiaries at an estimated cost of $3.4 billion (22). Cataracts were the cause of about 50% of cases of vision loss in white, African American, and Hispanic people (2). Women were somewhat more likely to be affected than men (21), but African American men were more likely to be blind from cataracts than white men (2). Variations in access to surgical treatment may account for some of the disparities. In east Baltimore, Md, blindness resulting from a cataract that had received no surgical treatment was four times more prevalent in African Americans than whites (23). In addition, the types of cataracts found most frequently in African Americans are different from those found in whites (24).

Because the lens naturally becomes more opaque over time, aging is the most important risk factor for developing cataracts. Although smoking has been implicated as a risk factor for developing nuclear cataracts, the conclusion of the Surgeon General's report on the health consequences of smoking is that the evidence is insufficient to conclude that smoking cessation reduces the risk for developing cataracts (7). Some studies revealed that a family history of cataracts and certain iris colors were risk factors, as were hypertension and diabetes (25). Increased exposure to sunlight, particularly ultraviolet B radiation, is also associated with an increased risk for developing cataracts (26). Surgical cataract removals are common, although local variations in surgical practice exist (22).

### Glaucoma

The incidence of glaucoma also increases with age. Risk factors other than age include a family history of glaucoma, African American ancestry, and diabetes (27). No significant sex differences were found in community studies (28). Vision loss in people with glaucoma is caused by a progressive loss of optic nerve fibers (27). Elevated intraocular pressure (IOP) is an important factor associated with loss of visual function, but not all patients with progressive vision loss from glaucoma experience consistently elevated pressure. Evaluating IOP is an important part of an eye examination when screening for glaucoma, but the diagnosis is based on detecting changes in the optic nerve and retinal nerve fibers during examination and by demonstrating decreased visual function, particularly in the periphery, using visual field testing. Most individuals with elevated IOP do not have optic nerve damage or changes in their visual fields, but studies have found that 1 in every 15 Americans with elevated IOP (higher than 21 mm Hg) has optic nerve changes typical of glaucoma (27). Treatments to decrease vision loss from the various pathophysiological processes that cause glaucoma have been well tested in numerous clinical trials (27). Because reducing elevated IOP is the mainstay of treatment to prevent vision loss, it is important to identify those with undiagnosed glaucoma before irreversible damage has occurred. In people aged 40 and older in the United States, the prevalence of glaucoma revealed by examination was 1.86%; the rate for African Americans was three times that for whites (28). However, these estimates do not include the numbers of people with elevated IOP (3). People with elevated IOP need careful treatment and follow-up examinations to prevent the development of irreversible vision loss (27).

## Impact of Age-related Eye Diseases on Public Health

The new prevalence estimates of AREDs, vision loss, and blindness in the United States have several important public health implications (2,3). First, a person often has more than one ARED, so aging individuals are at risk for multiple eye problems. Second, although The Eye Disease Prevalence Research Group studies demonstrated important disparities between African Americans and whites in the prevalence of individual AREDs, low vision, and blindness, the data were limited for several minority populations (2,3). The estimates were based on three eye studies that included African Americans; the prevalence of low vision and blindness among Hispanics was estimated from one study in southern Arizona (2,29). No information about American Indians or Asian Americans exists. Finally, little information is known about the way variable access to care or other factors may contribute to the striking variations in the prevalence of AREDs and loss of visual function. Investigators in east Baltimore, Md, estimated that half of the cases of blindness found in the community could have been prevented or reversed with proper care (23).

AREDs are common and costly. Treatment for cataracts accounted for 60% of the Medicare eye-care costs in the 1990s (30). In a longitudinal analysis of Medicare claims for diabetic retinopathy, glaucoma, and macular degeneration, investigators documented that almost half of more than 20,000 Medicare beneficiaries had developed at least one of the three diseases in the 9-year follow-up period (31). From the longitudinal data, the investigators concluded that among people who live to age 65 years, the probability of acquiring at least one of the three conditions is 0.45. Decreased vision is associated with myriad problems in older adults, such as falls, hip fractures, family stress, and depression (32). Vision disorders are also a safety risk to all automobile drivers and passengers (33).

Reducing the level of disability caused by vision loss and blindness is challenging for public health professionals for many reasons. For example, the magnitude of the problem is not completely understood because it is hard to measure vision loss and blindness accurately by the usual surveillance methods (34). People with limited eyesight may be less likely to have necessary examinations or answer surveys commonly used to assess chronic conditions. State-based blindness registries have not successfully documented the prevalence, risk factors, and trends in vision loss (34). In addition, to prevent vision loss, conditions such as glaucoma and diabetic retinopathy must be detected by careful and regular examinations *before* obvious symptoms of vision loss develop. Comprehensive dilated-eye examinations for these conditions are best performed by eye-care specialists — optometrists and ophthalmologists. It is easy for the general public to assume, erroneously, that a test of visual acuity associated with obtaining glasses or a driver's license will detect sight-threatening conditions. In addition, the geographical distribution of eye-care specialists is not known, and areas such as rural and medically underserved communities may have shortages. Finally, medical services to detect and treat serious eye diseases may not be integrated with the services needed to improve quality of life and increase independence for people with low vision. Glasses and other aids to improve vision are not uniformly covered by health insurance. Rehabilitation services to help people with vision loss are administered by agencies experienced in helping individuals return to work but are not usually linked with public health or home health services for older adults. Although *Healthy People 2010* (35) defines objectives for glaucoma, cataracts, and diabetic retinopathy, few communities have the resources to expand their efforts beyond diabetes-related eye disease (36). The recent estimates provided by The Eye Disease Prevalence Research Group not only highlight the lack of sufficient information about the prevalence of AREDs but also underscore the need for a better understanding of access to eye-care and vision support services among various populations.

## Conclusion

Public health professionals now have an additional responsibility — to better describe and, more importantly, address ARED and visual impairment prevalence disparities in the United States. The data provide a call to action. To decrease morbidity from vision loss and blindness, public health professionals must increase public awareness about AREDs; integrate and coordinate timely screening, diagnosis, and treatment to prevent or correct vision loss; ensure continuity of care between medical treatment and supportive care for vision loss from AREDs; and monitor the status of visual impairment by using new methods for identifying people affected and their extent of vision loss.
